# Impact of absolute lymphocyte count upon admission to ICU on 28-day mortality in sepsis patients in ICU

**DOI:** 10.3389/fimmu.2025.1688451

**Published:** 2026-01-05

**Authors:** DingJun Zhong, Ling Zhao

**Affiliations:** 1 Department of Clinical Medicine, XinJiang Medical University, Urumqi, China; 2 Department of Intensive Care Unit, The People’s Hospital Medical Group of Xiangzhou, Zhuhai, Guangdong, China

**Keywords:** sepsis, absolute lymphocyte count, 28-day mortality, prognostic prediction, Cox regression, MIMIC-IV database

## Abstract

**Background:**

Sepsis is associated with high mortality and poor prognosis in critically ill patients, posing a severe threat to global public health. The first absolute lymphocyte count (FALC) upon admission to the ICU, reflecting the initial state of the immune system, has emerged as a potential prognostic indicator in various critical illnesses. However, its specific role and predictive value in assessing 28-day mortality among sepsis patients in the ICU remain incompletely understood, with existing studies showing limitations in sample size and verification dimensions.

**Methods:**

Data of ICU patients (18<age<90) who met the sepsis diagnostic criteria (Sepsis-3 definition) were extracted from the MIMIC-IV(2.0) clinical database. Patients were grouped according to the tertiles of FALC (low, medium, high); FALC was the absolute lymphocyte count in the first complete blood count upon admission to the ICU; the outcome indicator was 28-day all-cause mortality. Baseline analysis: For measurement data, normally distributed data were described as mean ± standard deviation, skewed distributed data as median (Q1-Q3), and count data as frequency (percentage). Differences between groups were tested using t-test, chi-square test, or non-parametric test. Survival analysis: KM method was used to draw survival curves, and log-rank test was used to compare differences between groups; Cox proportional hazards model was used to analyze univariate/multivariate associations, and hazard ratios (HR) and 95% confidence intervals (CI) were calculated. Non-linear verification: RCS model was used to fit the non-linear relationship between FALC and death risk, and the linear hypothesis was tested.

**Predictive efficacy:**

Cox regression ROC curve was used to calculate the area under the curve (AUC) to evaluate the predictive value of FALC.

**Results:**

The study of 10,263 sepsis patients admitted to the ICU found that FALC grouping significantly affected 28-day survival outcomes, as shown by Kaplan-Meier survival analysis and Cox regression analysis. Low FALC group having a significantly higher 28-day mortality risk compared to the medium and high groups. Restricted cubic splines verified a non-linear relationship between FALC and 28-days mortality, and the ROC curve confirmed that FALC has a certain prognostic predictive efficacy.

**Conclusion:**

In sepsis patients, lymphopenia represents a significant high-risk factor, while the first absolute lymphocyte count (FALC) stands out as a clinically meaningful indicator. It allows for precise risk stratification of patients at the time of ICU admission, thereby enabling timely immune interventions that can effectively enhance prognostic outcomes.

## 1 Introduction

Sepsis, as an extremely challenging acute and critical illness in ICU clinical diagnosis and treatment, has become one of the leading causes of death in critically ill patients (1, 2). Globally, millions of patients are afflicted by this dangerous disease every year, and their lives and health are continuously under severe threat. According to data from multiple authoritative clinical studies, the mortality rate of sepsis patients remains high. The average 30-day mortality rate in patients with sepsis was 24.4% (95% CI 21.5%–27.2%), and the average 90-day mortality rate was 32.2% (95% CI 27.0%–37.5%) (3). Even with the continuous advancement of medical technology, the mortality rate of severe sepsis and septic shock patients remains at a high level, bringing a heavy burden to patients’ families and society (4).

Given the characteristics of rapid progression and significant prognostic differences of sepsis, early and accurate prognostic assessment is particularly crucial. It is not only an important prerequisite for formulating individualized treatment decisions but also a core link in improving patients’ clinical outcomes. Through scientific and effective prognostic assessment, clinicians can promptly identify high-risk patients, thereby taking targeted intervention measures earlier, optimizing treatment plans, minimizing the risk of death, and improving patients’ survival rate and quality of life (5, 6).

Lymphocytes, as a core component of the body’s immune system, play an irreplaceable role in resisting pathogen invasion and maintaining immune homeostasis (7). The dynamic changes in their quantity, especially measured by the first absolute lymphocyte count (FALC) upon admission to the ICU, have attracted extensive attention in academic and clinical fields in recent years due to their close association with immune dysfunction and disease prognosis in sepsis patients (8). A large number of studies have shown that when sepsis occurs, the body’s immune system will experience disorders such as excessive activation or immune suppression, and the quantity and function of lymphocytes will often undergo significant changes. A decrease in FALC may indicate impaired immune function, which is closely related to the severity of the patient’s condition and poor prognosis (9).

However, despite the existing studies on the association between FALC and sepsis prognosis, these studies still have obvious limitations in many aspects. In terms of sample size, most studies are limited to small-scale single-center data, lacking the support of large-scale, multi-center large-sample studies, which affects the representativeness and persuasiveness of the research results. In terms of verification dimensions, existing studies mostly focus on the analysis of a single indicator or a single outcome, and there is a lack of multi-dimensional and multi-level verification, such as comprehensive analysis combined with patients’ clinical characteristics, other immune indicators, treatment responses, etc. It is difficult to comprehensively and deeply reveal the complex relationship between FALC and sepsis prognosis, which also restricts its wide application and promotion in clinical practice.

## 2 Materials

### 2.1 Population

The MIMIC-IV database was established with funding from the NIH by a team of multidisciplinary experts from Beth Israel Deaconess Medical Center (BIDMC), the Massachusetts Institute of Technology (MIT), the University of Oxford, and Massachusetts General Hospital (MGH). It contains clinical data from over 190,000 patients and 450,000 hospitalization records admitted to BIDMC between 2008 and 2019, encompassing rich patient diagnosis and treatment information, thus providing sufficient data support for various medical research endeavors (10).

**Figure 1:**
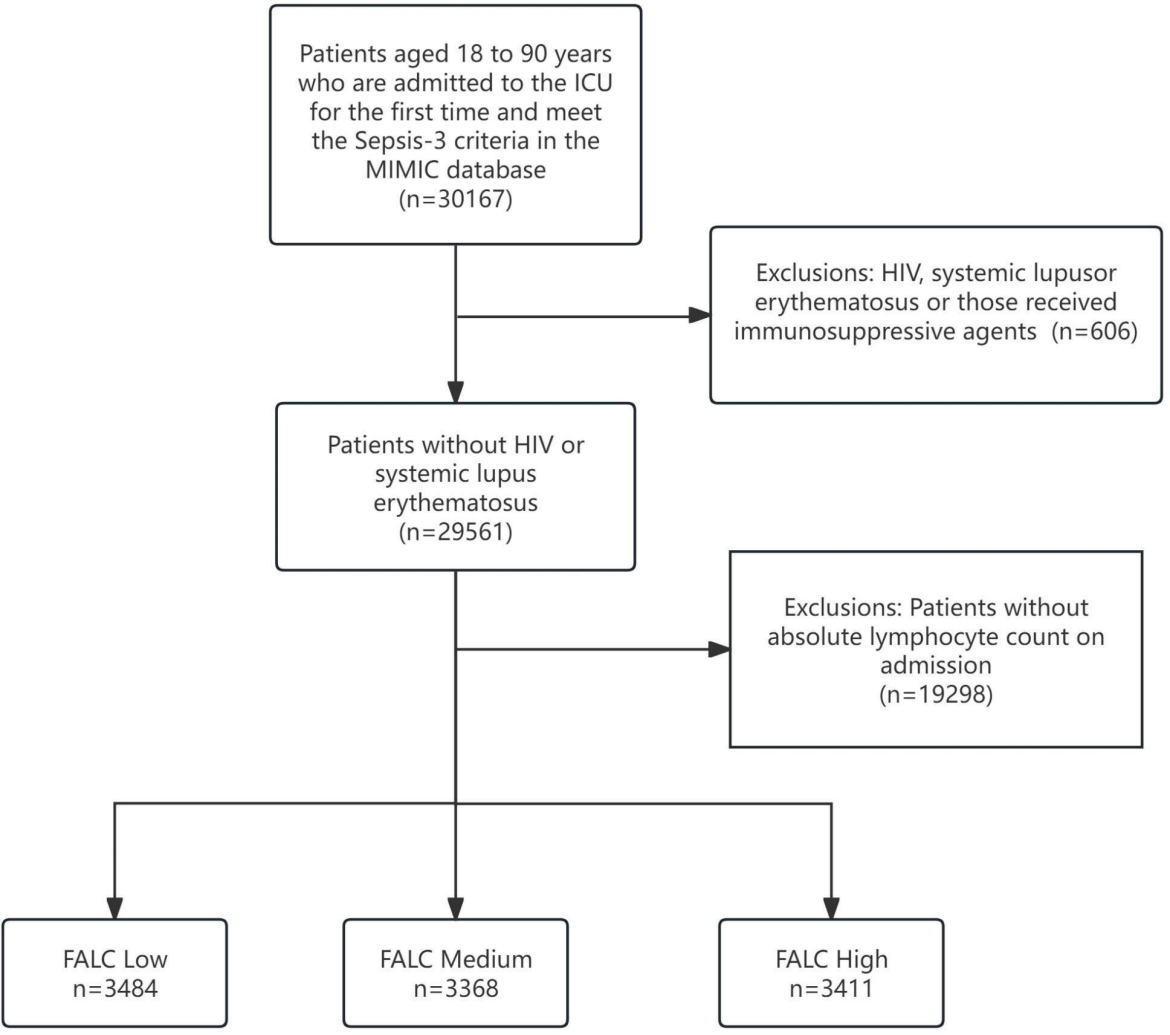
Flowchart of the study.

In the database, there were 30,167 patients aged 18 to 90 years who were admitted to the ICU for the first time and met the Sepsis-3 criteria. Among them, 606 patients had HIV, systemic lupus erythematosus or those received immunosuppressive agents were excluded. Subsequently, we excluded patients lacking the first absolute lymphocyte count (FALC) upon admission (**Figure 1**). Finally, 10,263 eligible patients were included in the study. Then we grouped according to the tertiles of absolute lymphocyte count (FALC) upon admission to the ICU, the low, medium, and high FALC groups.

### 2.2 Data collection

A critical step in ensuring the validity of associations between FALC and 28-day mortality involves accounting for confounding variables. Based on the presented dataset, the following factors were identified as potential confounders, as they may independently associate with both immune status, reflected by FALC, and sepsis prognosis: Age, weight, SOFA, white blood cells, hemoglobin, neutrophils, platelet count, potassium, lactate, urea nitrogen, creatinine, gender. Regarding comorbidities, we considered the presence or absence of acute kidney injury (AKI), chronic kidney disease (CKD), chronic obstructive pulmonary disease (COPD), heart failure (HF), hyperlipidemia, hypertension, liver cirrhosis, and type 1 and type 2 diabetes mellitus.

The follow-up period starts from the moment the patient is admitted to the ICU and continues until death or 28 days have elapsed.

Data was extracted via PostgreSQL (version 13.7.2) and Navicat Premium (version 16) through the execution of Structured Query Language (SQL) commands.

For missing data, we employed imputation methods: median imputation was used for continuous variables, and mode imputation for categorical variables. Detailed counts and rates of missing data are provided in **Supplementary Material 1**.

### 2.3 Clinical outcomes

The main result of this study was the all-cause mortality rate at 28 days following hospital admission.

### 2.4 Analysis of statistics

For the data, we used the Kolmogorov-Smirnov test method to perform a univariate normality test on each variable. The results include P-values and an indication of whether the variable follows a normal distribution. If the P-value is greater than 0.05, the variable is considered to follow a normal distribution. The results indicated that all variables were skewed distributed, so they were described using median (P25-P75).

We conducted a baseline analysis on the collected data and used p-values for hypothesis testing to determine whether there were significant differences between multiple groups. The methods employed were the Kruskal-Wallis Rank Sum Test and Pearson’s Chi-Squared Test.

Following the completion of the baseline analysis, we further generated Kaplan-Meier curves using the Log-Rank Test to visually demonstrate the dynamic changes in survival differences over time between different groups.

We conducted univariate Cox regression analyses on each potential variable identified from the baseline analysis, calculating the hazard ratio (HR) and 95% confidence interval for each; statistical significance was defined as a p-value < 0.001. Following this, multivariate Cox regression was performed to adjust for confounding factors and validate the robustness of key variables.

After grouping according to the tertiles of FALC (Low, Medium, High), the grouping was significantly associated with death risk: HR = 0.631 (95% CI: 0.572 - 0.697, P < 0.001) in the Medium group, and HR = 0.46 (95% CI: 0.412 - 0.512, P < 0.001) in the High group. However, when FALC was treated as a continuous variable, the Cox regression analysis showed a p-value of 0.722. This suggests that there may be a non-linear relationship between FALC and death, which requires further analysis.

To verify the presence of a non-linear relationship, we used restricted cubic spline plots for validation and concluded that there is indeed a non-linear relationship between FALC and mortality.

## 3 Results

We enrolled 10,263 sepsis patients aged 18 to 90 years. Among them, males accounted for 61.93% (6,356 cases), and females accounted for 38.07%. The median age was 66 years (interquartile range [IQR], 58–76 years), with a median body weight of 83.20 kg (IQR, 69.20–94.80 kg). The median SOFA score was 6.00 (IQR, 4.00–8.00).

For laboratory indicators, the median first white blood cell count was 12.20 × 10^9/L (IQR, 8.70–16.70 × 10^9/L); the median hemoglobin level was 10.00 g/dL (IQR, 8.60–11.70 g/dL); the median percentage of first neutrophils was 79.80% (IQR, 72.80–85.30%); the median first platelet count was 171.00 × 10^9/L (IQR, 120.00–237.00 × 10^9/L); the median first potassium level was 4.20 mEq/L (IQR, 3.80–4.60 mEq/L); the median first lactate level was 1.90 mmol/L (IQR, 1.40–2.70 mmol/L); the median first urea nitrogen level was 21.00 mg/dL (IQR, 15.00–35.00 mg/dL); and the median first creatinine level was 1.10 mg/dL (IQR, 0.80–1.70 mg/dL).

The number of deaths within 28 days was 2,125, with a 28-day mortality rate of 20.71%.

Regarding comorbidities, 5,424 (52.85%) patients had acute kidney injury (AKI), 1,116 (10.87%) had liver cirrhosis, 2,416 (23.54%) had chronic kidney disease (CKD), 3,235 (31.52%) had type 2 diabetes mellitus (T2DM), 188 (1.83%) had type 1 diabetes mellitus (T1DM), 4,197 (40.89%) had hyperlipidemia, 3,228 (31.45%) had heart failure (HF), and 1,702 (16.58%) had chronic obstructive pulmonary disease (COPD).

Patients in the lowest FALC quartile exhibited a higher 28-day mortality rate (p < 0.001), reported higher SOFA scores (p < 0.001), increased white blood cell counts (p < 0.001) as well as higher levels of lactate (p < 0.001), urea nitrogen (p < 0.001) and creatinine (p < 0.001) compared to those in the higher quartiles (**Table 1**).

**Table 1:** Baseline characteristics.

Variable	Levels	N	Overall	Low	Medium	High	p-value
			N = 10,263	N = 3,484	N = 3,368	N = 3,411	
FALC. median(10^9/L)		10263	1.06 (0.61 - 1.69)	0.46 (0.30 - 0.62)	1.07 (0.90 - 1.24)	2.04 (1.70 - 2.66)	<0.001
Age		10263	66.00 (58.00 - 76.00)	67.00 (60.00 - 76.00)	66.00 (57.00 - 76.00)	65.24 (56.00 - 75.00)	<0.001
weight (kg)		10263	83.20 (69.20 - 94.80)	81.20 (66.80 - 91.80)	82.80 (69.16 - 94.28)	84.15 (71.50 - 98.20)	<0.001
icu_survival_time (days)		10263	28.00 (28.00 - 28.00)	28.00 (18.39 - 28.00)	28.00 (28.00 - 28.00)	28.00 (28.00 - 28.00)	<0.001
SOFA		10263	6.00 (4.00 - 8.00)	6.51 (4.00 - 9.00)	6.00 (4.00 - 8.00)	6.00 (4.00 - 8.00)	<0.001
first_white_blood_cells (10^9/L)		10263	12.20 (8.70 - 16.70)	11.20 (6.70 - 14.80)	12.20 (8.80 - 16.50)	13.20 (10.40 - 18.30)	<0.001
first_hemoglobin (g/dL)		10263	10.00 (8.60 - 11.70)	10.00 (8.40 - 11.40)	10.00 (8.70 - 11.90)	10.00 (8.70 - 11.70)	<0.001
first_neutrophils (%)		10263	79.80 (72.80 - 85.30)	84.70 (79.50 - 90.00)	79.90 (75.10 - 84.90)	74.80 (67.10 - 79.80)	<0.001
first_platelet_count (10^9/L)		10263	171.00 (120.00 - 237.00)	167.00 (102.00 - 223.00)	171.00 (129.00 - 245.00)	171.00 (129.00 - 241.00)	<0.001
first_potassium (mEq/dL)		10263	4.20 (3.80 - 4.60)	4.20 (3.80 - 4.60)	4.20 (3.80 - 4.60)	4.20 (3.90 - 4.60)	0.016
first_lactate (mmol/L)		10263	1.90 (1.40 - 2.70)	1.90 (1.40 - 2.80)	1.90 (1.40 - 2.50)	1.90 (1.40 - 2.70)	<0.001
first_urea_nitrogen (mg/dL)		10263	21.00 (15.00 - 35.00)	25.00 (17.00 - 43.00)	21.00 (14.00 - 34.00)	19.00 (13.00 - 28.00)	<0.001
first_creatinine (mg/dL)		10263	1.10 (0.80 - 1.70)	1.10 (0.90 - 1.90)	1.10 (0.80 - 1.70)	1.00 (0.80 - 1.40)	<0.001
first_albumin, median (p25 - p75)		10263	2.90 (2.70 - 3.10)	2.90 (2.60 - 3.10)	2.90 (2.70 - 3.20)	2.90 (2.90 - 3.10)	<0.001
gender, n (p%)		10263					0.119
	F		3,907.00 (38.07%)	1,281.00 (36.77%)	1,291.00 (38.33%)	1,335.00 (39.14%)	
	M		6,356.00 (61.93%)	2,203.00 (63.23%)	2,077.00 (61.67%)	2,076.00 (60.86%)	
death_within_icu_28days, n (p%)		10263					<0.001
	0		8,138.00 (79.29%)	2,490.00 (71.47%)	2,722.00 (80.82%)	2,926.00 (85.78%)	
	1		2,125.00 (20.71%)	994.00 (28.53%)	646.00 (19.18%)	485.00 (14.22%)	
Hypertension, n (p%)		10263					<0.001
	0		6,664.00 (64.93%)	2,439.00 (70.01%)	2,196.00 (65.20%)	2,029.00 (59.48%)	
	1		3,599.00 (35.07%)	1,045.00 (29.99%)	1,172.00 (34.80%)	1,382.00 (40.52%)	
Acute kidney injury, n (p%)		10263					<0.001
	0		4,839.00 (47.15%)	1,350.00 (38.75%)	1,604.00 (47.62%)	1,885.00 (55.26%)	
	1		5,424.00 (52.85%)	2,134.00 (61.25%)	1,764.00 (52.38%)	1,526.00 (44.74%)	
Liver cirrhosis, n (p%)		10263					<0.001
	0		9,147.00 (89.13%)	2,997.00 (86.02%)	2,995.00 (88.93%)	3,155.00 (92.49%)	
	1		1,116.00 (10.87%)	487.00 (13.98%)	373.00 (11.07%)	256.00 (7.51%)	
Chronic kidney disease, n (p%)		10263					<0.001
	0		7,847.00 (76.46%)	2,528.00 (72.56%)	2,612.00 (77.55%)	2,707.00 (79.36%)	
	1		2,416.00 (23.54%)	956.00 (27.44%)	756.00 (22.45%)	704.00 (20.64%)	
Type 2 diabetes mellitus, n (p%)		10263					0.064
	0		7,028.00 (68.48%)	2,409.00 (69.14%)	2,335.00 (69.33%)	2,284.00 (66.96%)	
	1		3,235.00 (31.52%)	1,075.00 (30.86%)	1,033.00 (30.67%)	1,127.00 (33.04%)	
Type 1 diabetes mellitus, n (p%)		10263					0.131
	0		10,075.00 (98.17%)	3,411.00 (97.90%)	3,303.00 (98.07%)	3,361.00 (98.53%)	
	1		188.00 (1.83%)	73.00 (2.10%)	65.00 (1.93%)	50.00 (1.47%)	
Hyperlipidemia, n (p%)		10263					<0.001
	0		6,066.00 (59.11%)	2,175.00 (62.43%)	2,063.00 (61.25%)	1,828.00 (53.59%)	
	1		4,197.00 (40.89%)	1,309.00 (37.57%)	1,305.00 (38.75%)	1,583.00 (46.41%)	
Heart failure, n (p%)		10263					<0.001
	0		7,035.00 (68.55%)	2,298.00 (65.96%)	2,315.00 (68.74%)	2,422.00 (71.01%)	
	1		3,228.00 (31.45%)	1,186.00 (34.04%)	1,053.00 (31.26%)	989.00 (28.99%)	
COPD, n (p%)		10263					<0.001
	0		8,561.00 (83.42%)	2,800.00 (80.37%)	2,804.00 (83.25%)	2,957.00 (86.69%)	
	1		1,702.00 (16.58%)	684.00 (19.63%)	564.00 (16.75%)	454.00 (13.31%)	

0 indicates a negative result

1 indicates a positive result

### 3.1 Survival analysis

**Figure 2:**
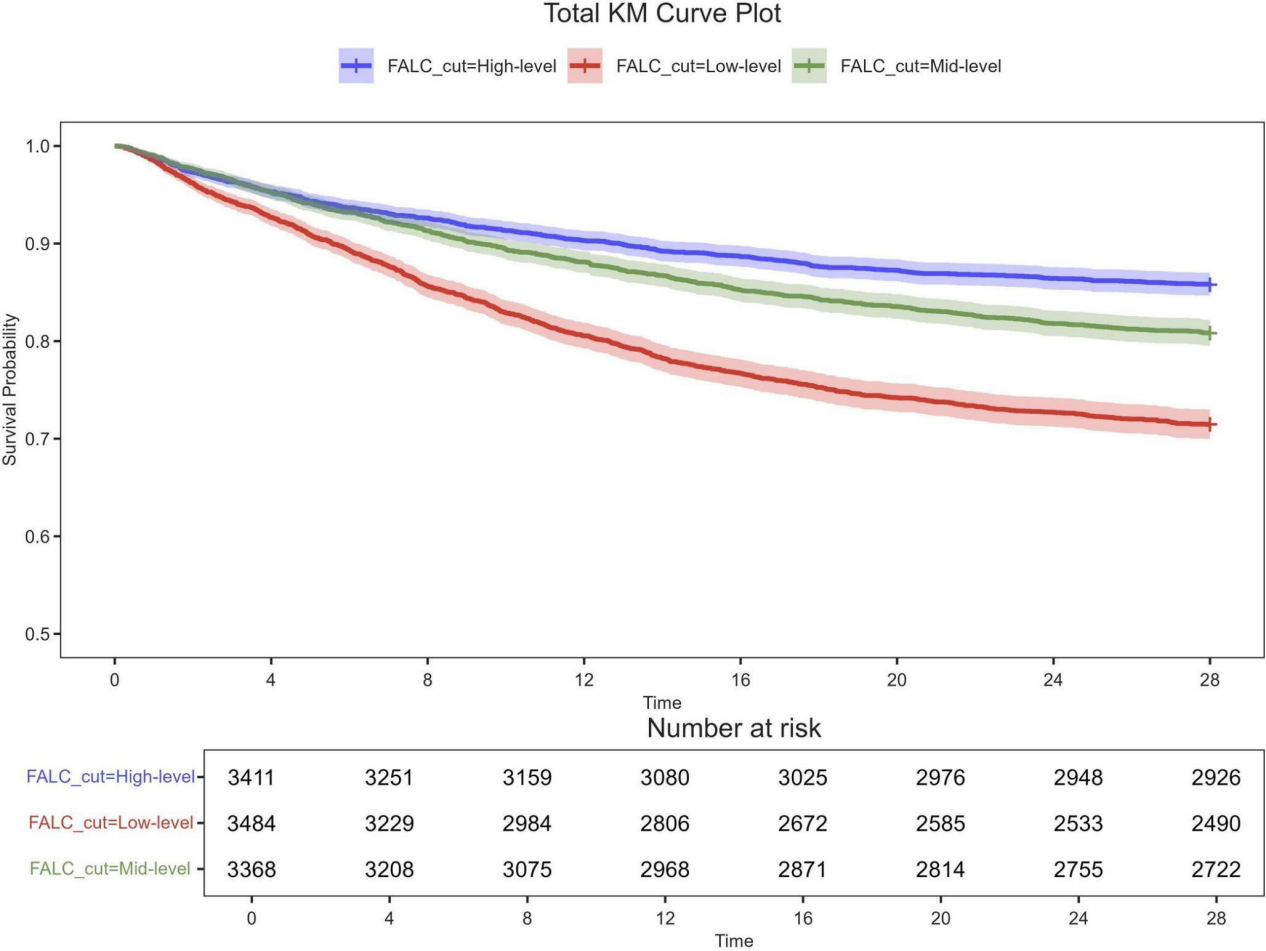
Kaplan-Meier (KM) survival curve plot for all variables, depicting overall survival rates over time (vertical axis : survival rate; horizontal axis: timeline) with confidence intervals (shaded). The below table includes proportional hazards adjustment. Continuous variables were grouped by median cutoff.

The KM curve showed: the 28-day survival rate of the low FALC group was significantly lower than that of the medium and high groups (log-rank P < 0.001), and the high FALC group had the most obvious survival advantage, suggesting that the lower the FALC, the higher the risk of death (**Figure 2**).

To explore the risk factors for predicting 28-day mortality in sepsis patients, univariate Cox regression analysis was performed. In the univariate analysis, age (p < 0.001), Sequential Organ Failure Assessment (SOFA) score (p < 0.001), initial peripheral blood indicators (absolute lymphocyte count grouping, white blood cell count, neutrophil count, lactic acid, creatinine) and Acute kidney injury, Chronic kidney disease, COPD, Heart failure, Hypertension, Liver cirrhosis with p-values < 0.001 were significantly associated with 28-day mortality.

**Figure 3:**
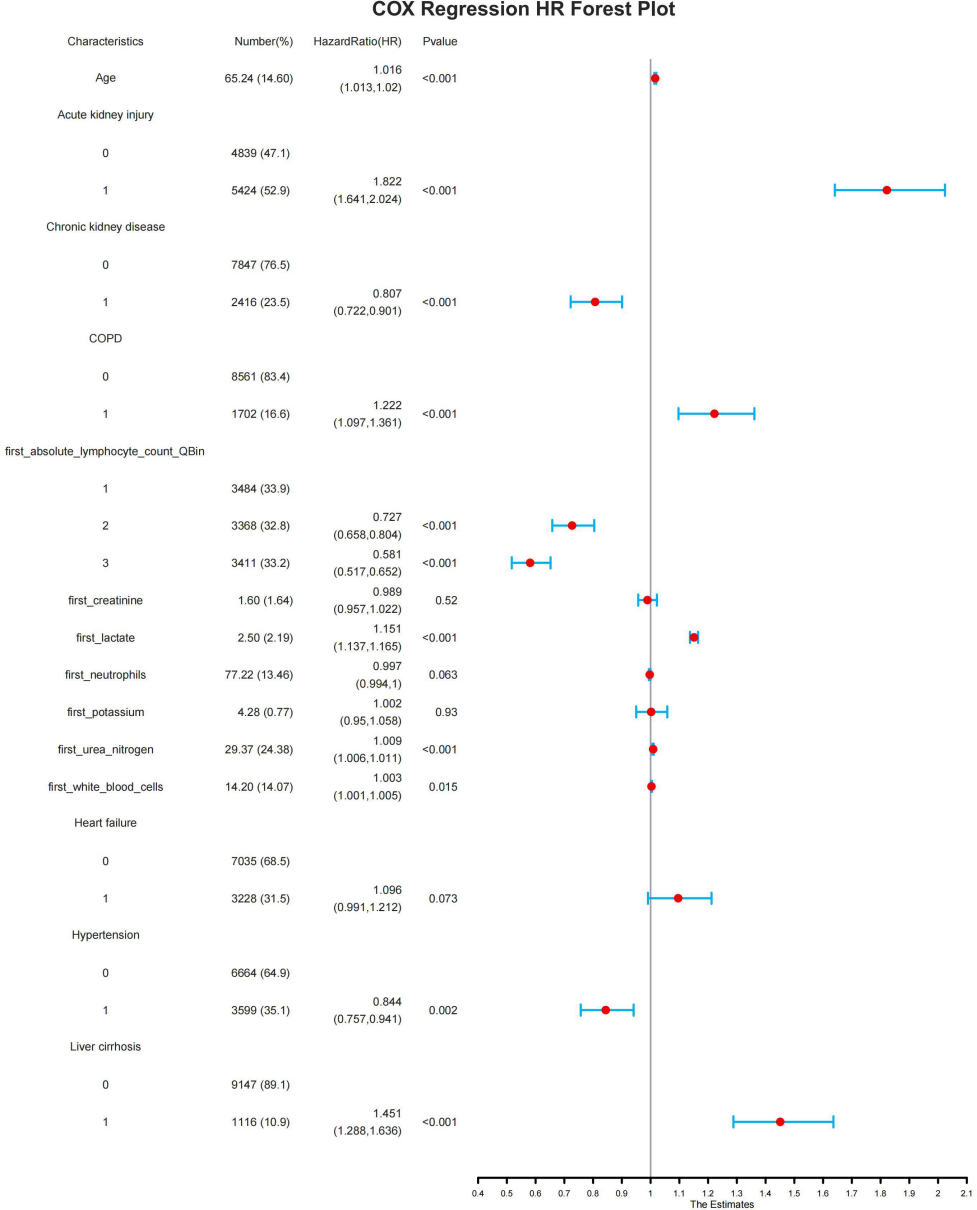
This figure is a multivariate Cox regression hazard ratio (HR) forest plot. It displays all the variables of the factors, along with their hazard ratios and corresponding 95% confidence intervals (Cls).

Multivariate analysis showed that age, absolute lymphocyte count grouping, creatinine, lactic acid, urea nitrogen, SOFA score (p < 0.001) and Acute kidney injury, Chronic kidney disease, COPD, Liver cirrhosis, were independent risk factors for poor 28-day survival prognosis in sepsis patients (**Table 2**). A forest plot of hazard ratios (HR) from the multivariate Cox regression analysis displays all the variables of the factors, along with their hazard ratios and corresponding confidence intervals. The vertical line in the middle represents the null line. If the horizontal line of the interval crosses this vertical line, it indicates that the factor has no association with the outcome. If the horizontal line of the interval lies to the left of the vertical line, the factor is favorable for the occurrence of the outcome. If the horizontal line of the interval is to the right of the vertical line, the factor is unfavorable for the occurrence of the outcome (**Figure 3**).

**Table 2:** Univariate and multivariate Cox regression.

Characteristics		Univariable				Multivariable		
	Hazard ratio (HR)	lower_95	upper_95	Pvalue	Hazard ratio (HR)	lower_95	upper_95	Pvalue
Age	1.018	1.015	1.021	<0.001	1.016	1.013	1.02	<0.001
Acute kidney injury								
0								
1	2.719	2.469	2.994	<0.001	1.822	1.641	2.024	<0.001
Chronic kidney disease								
0								
1	1.364	1.242	1.499	<0.001	0.807	0.722	0.901	<0.001
COPD								
0								
1	1.332	1.199	1.48	<0.001	1.222	1.097	1.361	<0.001
FALC	0.999	0.993	1.005	0.722				
first_absolute_lymphocyte_count_QBin								
Low								
Medium	0.631	0.572	0.697	<0.001	0.727	0.658	0.804	<0.001
High	0.46	0.412	0.512	<0.001	0.581	0.517	0.652	<0.001
first_creatinine	1.101	1.083	1.118	<0.001	0.989	0.957	1.022	0.52
first_hemoglobin	0.972	0.954	0.99	0.003				
first_lactate	1.177	1.163	1.19	<0.001	1.151	1.137	1.165	<0.001
first_neutrophils	1.008	1.004	1.011	<0.001	0.997	0.994	1	0.063
first_platelet_count	1	0.999	1	0.373				
first_potassium	1.211	1.15	1.275	<0.001	1.002	0.95	1.058	0.93
first_urea_nitrogen	1.012	1.011	1.013	<0.001	1.009	1.006	1.011	<0.001
first_white_blood_cells	1.004	1.002	1.005	<0.001	1.003	1.001	1.005	0.015
Gender								
F								
M	0.916	0.84	1	0.049				
Heart failure								
0								
1	1.433	1.313	1.563	<0.001	1.096	0.991	1.212	0.073
Hyperlipidemia								
0								
1	0.928	0.85	1.012	0.091				
Hypertension								
0								
1	0.741	0.675	0.814	<0.001	0.844	0.757	0.941	0.002
Liver cirrhosis								
0								
1	1.849	1.653	2.069	<0.001	1.451	1.288	1.636	<0.001
SOFA	1.189	1.176	1.201	<0.001	1.147	1.133	1.161	<0.001
Type 1 diabetes mellitus								
0								
1	0.718	0.498	1.036	0.077				
Type 2 diabetes mellitus								
0								
1	1.047	0.956	1.146	0.326				
Weight	0.999	0.997	1.001	0.353				

0 indicates a negative result

1 indicates a positive result

**Figure 4:**
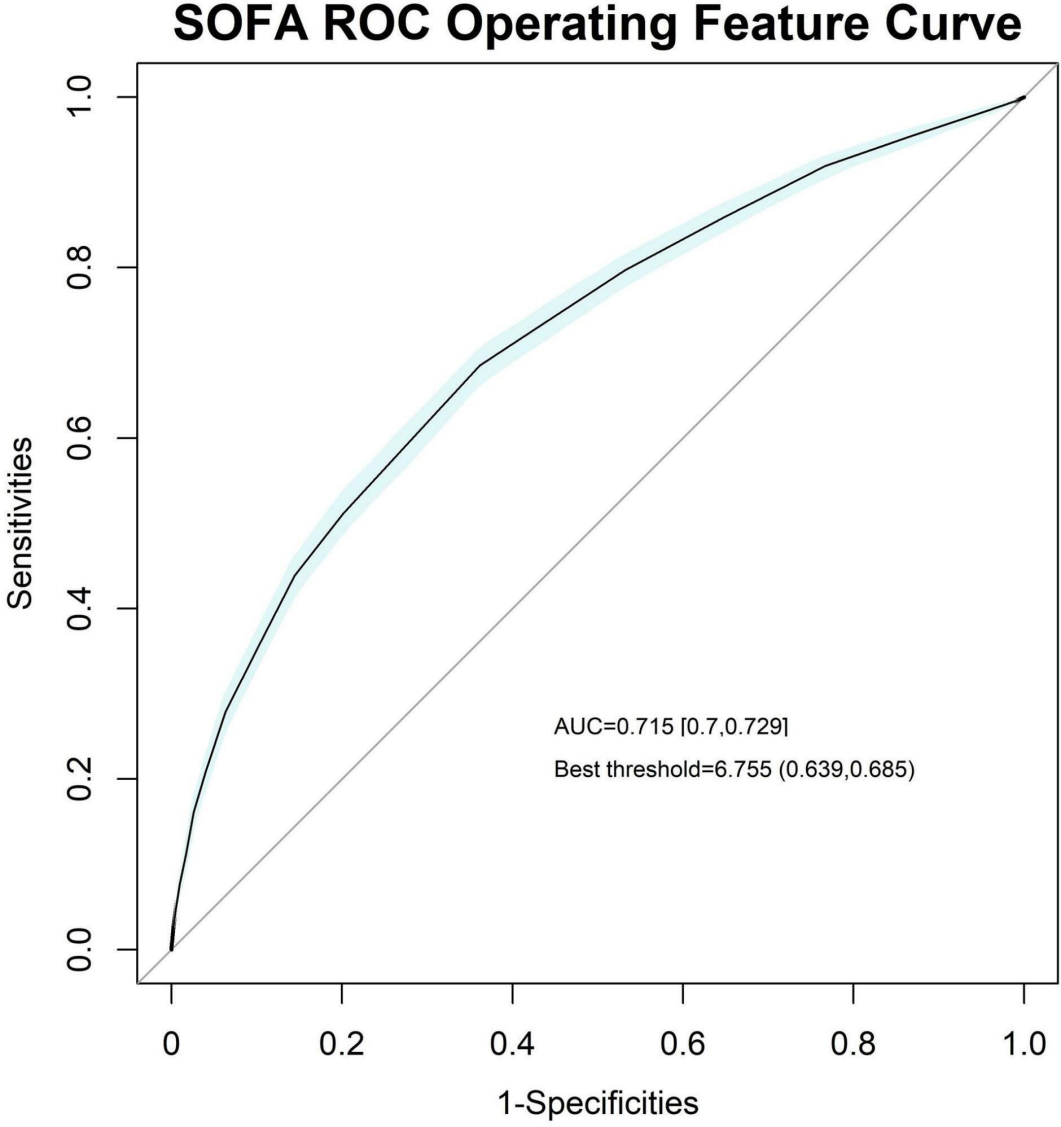
Receiver operating characteristic (ROC) curve of the SOFA score, with an area under the curve (AUC) of 0.715 [0.7, 0.729] and a best threshold of 6.755.

**Figure 5:**
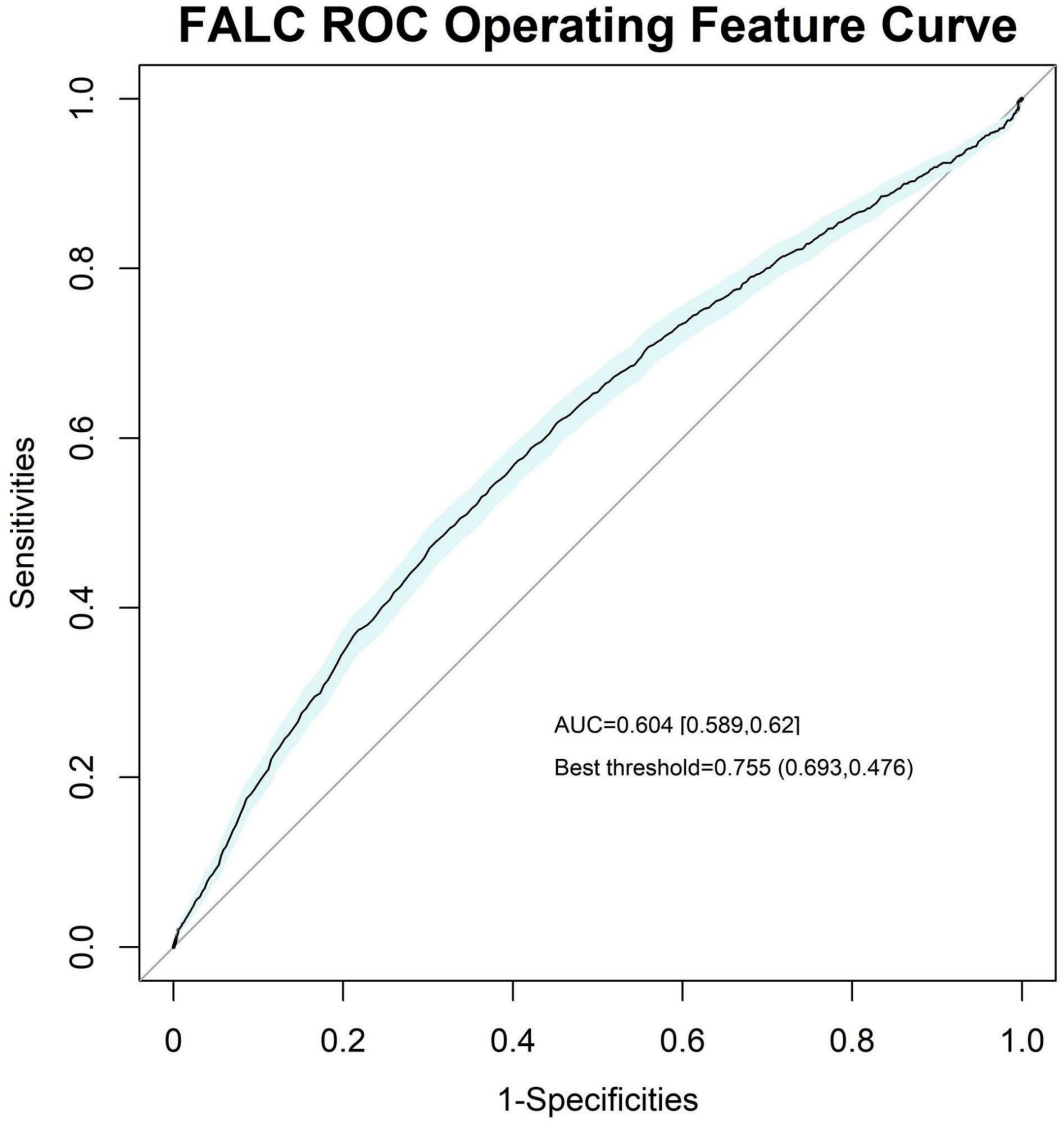
Receiver operating characteristic (ROC) curve of the FALC, with an area under the curve (AUC) of 0.604 [0.589, 0.621] and a best threshold of 0.755.

We also plotted the ROC curves of SOFA and FALC to compare and examine the effect of FALC (**Figures 4**, **5**).

It is worth noting that when FALC was a continuous variable, its association with 28-day death risk in the univariate analysis was not statistically significant (HR = 0.999 [0.993-1.005] p = 0.722).

**Figure 6:**
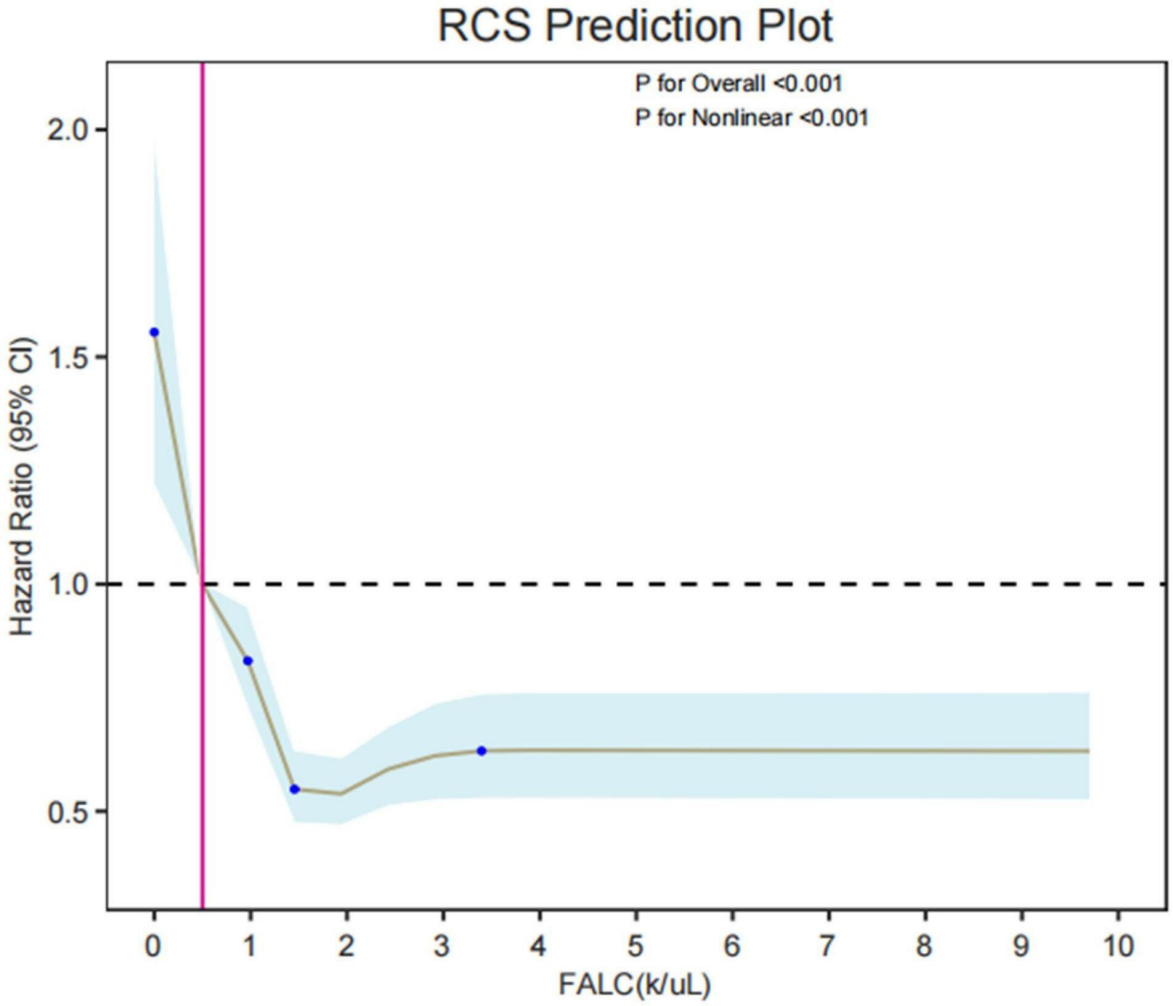
Restricted cubic spline (RCS) prediction plot showing the nonlinear relationship between FALC (cells/uL) and hazard ratio (95% CI), with both overall (P < 0.001) and nonlinear (P < 0.001) effects being statistically significant.

Verification of non-linear relationship (RCS model) The RCS curve showed (**Figure 6**): FALC was non-linearly and negatively correlated with death risk (P for Nonlinear < 0.001). When FALC < 1×10^9^/L, the death risk increased sharply with the decrease of FALC; after FALC > 1×10^9^/L, the risk tended to be stable. When FALC < 0.5×10^9^/L, there was a risk of death. It further confirmed that the protective effect of FALC on death risk has a threshold effect and is a non-linear relationship.

## 4 Discussion

In this large-scale retrospective study utilizing the MIMIC-IV(2.0) database, we systematically demonstrated that the first absolute lymphocyte count (FALC) upon ICU admission is an independent and robust predictor of 28-day mortality in sepsis patients. Through multi-dimensional statistical validations, including Kaplan-Meier survival analysis, univariate/multivariate Cox regression, restricted cubic splines (RCS), and Cox regression ROC curves, we identified a non-linear negative association between FALC and mortality risk: when FALC is below 1×10^9^/L, the risk of death increases sharply; beyond this threshold, the risk stabilizes, highlighting a critical “threshold effect” in its prognostic value. This finding aligns with the pathophysiological mechanisms of sepsis-induced immune dysfunction—specifically, the profound immune suppression characterized by excessive lymphocyte apoptosis, immune exhaustion, and impaired T-cell function (11). Such mechanisms directly contribute to the observed lymphopenia, as sepsis triggers a cascade of pro-inflammatory cytokines that disrupt lymphocyte homeostasis, while mitochondrial dysfunction and oxidative stress further accelerate lymphocyte loss (12, 13). Notably, our data clarify that the relationship between FALC and sepsis outcomes is not simply linear, a nuance overlooked in previous small-sample studies, thereby offering novel insights into how immune cell depletion drives poor prognosis in sepsis.

Consistent with a recent study by Huang et al. (2025), which also leveraged the MIMIC-IV database to explore the prognostic role of absolute lymphocyte count (ALC) in sepsis, our findings confirm that lymphocyte count (as FALC in our research) is an independent predictor of mortality—their multivariate Cox analysis reported an HR of 0.88 (P < 0.001) for ALC, while our univariate analysis yielded an HR of 0.62 (P < 0.001) for FALC, both underscoring the protective effect of higher lymphocyte counts. However, Huang et al. focused on integrating ALC into the SOFA score to enhance prognostic accuracy, demonstrating improved area under the receiver operating characteristic curve for 28-day (0.680 vs. 0.664) and 90-day (0.666 vs. 0.647) mortality compared to the original SOFA score. In contrast, our study uniquely emphasizes the non-linear “threshold effect” of FALC (1×10^9^/L), a nuance not explicitly addressed in their ALC-quartile-based scoring system. While both studies validate ALC’s clinical utility, ours provides a precise risk stratification threshold, whereas the ALC-SOFA score offers a comprehensive organ+immune integrated tool—highlighting the complementary value of ALC in sepsis prognosis. Additionally, Huang et al. noted that ALC-SOFA’s predictive advantage was absent in immunocompromised patients, which aligns with our recognition of the need for subgroup-specific validation of FALC’s utility (14).

Our focus on admission FALC as a prognostic marker also complements Chen et al.’s (2024) retrospective big data study, which emphasized the predictive superiority of dynamic ALC changes—specifically, day 7 ALC had the highest AUC for 90-day mortality (cut-off value: 1.0×10^9^/L) and outperformed the SOFA score in severely ill (SOFA ≥ 6) and young (<60 years) patients. Chen et al. highlighted that dynamic ALC trajectories better reflect immune suppression kinetics, a point we also acknowledge in our future research directions. However, our study identifies that a single admission FALC measurement still holds robust predictive value for 28-day mortality, with a consistent threshold (1×10^9^/L) to that of Chen et al.’s day 7 ALC cut-off—suggesting that both early static and late dynamic ALC measurements converge on a critical lymphopenia threshold for poor outcomes. Notably, Chen et al. used multiple machine learning models to validate ALC’s additive value, while our RCS analysis reveals the non-linear nature of FALC’s association with mortality—an aspect not fully explored in their linear HR-based analysis. Together, these studies demonstrate that ALC’s prognostic power is retained across both static (admission) and dynamic (longitudinal) measurements, with our work specifying the early threshold effect and Chen et al. emphasizing long-term trajectory value (8).

Clinically, the non-linear predictive value of FALC carries substantial implications for optimizing sepsis management, particularly in addressing immune suppression. For patients with FALC < 1×10^9^/L early intervention should prioritize reversing immune paralysis: this includes monitoring T-cell subsets (e.g., CD4^+^/CD8^+^ ratios) to quantify immune depletion, considering administering immunomodulators to restore T-cell function (15–17). In contrast, patients with medium to high FALC may retain relatively intact immune competence, making aggressive infection source control and targeted anti-infective therapy more critical to avoid exacerbating immune suppression through overtreatment (18, 19). As a readily accessible and cost-effective biomarker, FALC bridges the gap between bench and bedside, enabling clinicians to stratify patients based on immune status and tailor interventions to preserve or restore immune homeostasis—ultimately improving overall survival and reducing the burden of severe sepsis (20, 21).

Future research should focus on several directions to translate these findings into broader clinical practice, with a particular emphasis on immune mechanisms. First, prospective cohort studies tracking dynamic changes in FALC are warranted, as such trajectories may better reflect the kinetics of immune suppression—including the transition from hyperinflammation to immune exhaustion—and enhance prognostic accuracy. Meanwhile, there are also studies elaborating that lymphopenia in sepsis patients, as a dynamic indicator, can better predict the condition and prognosis of sepsis patients (22, 23). Second, integrating FALC with other immune-related indicators such as neutrophil-to-lymphocyte ratio, procalcitonin, or cytokine profiles could establish more comprehensive models to capture the complexity of immune dysregulation, addressing the limitations of single-marker prediction (24–26). Third, subgroup analyses in specific populations are needed to explore how immune cell dynamics vary across contexts and refine FALC’s predictive utility. Finally, interventional studies targeting FALC-related immune mechanisms—such as evaluating whether enhancing lymphocyte survival or restoring T-cell function reduces mortality—will help validate the causal link between lymphopenia, immune suppression, and sepsis outcomes, promoting the translation of basic immunology research into clinical benefits. Fourth, continuous monitoring of absolute lymphocyte count (ALC) may offer advantages over a single admission-only measurement. Dynamic tracking of ALC changes enables real-time reflection of fluctuations in immune status, such as the progression of immunosuppression, therapeutic response, and risk of exhaustion, thereby providing more precise temporal evidence for individualized interventions and addressing the limitations of single measurements.

## 5 Conclusion

FALC upon ICU admission represents a promising tool for early risk stratification and treatment guidance in sepsis, with its non-linear association with mortality directly reflecting the dynamics of immune suppression and lymphocyte depletion. Its clinical utility underscores the importance of individualized immune assessment.

## Data Availability

Publicly available datasets were analyzed in this study. This data can be found here: https://mimic.physionet.org.
